# Velocity‐Dependent Stress Changes in the Hamstring Muscle‐Tendon Complex: A Study Using Human Cadavers

**DOI:** 10.1155/tsm2/4876843

**Published:** 2026-04-21

**Authors:** Gakuto Nakao, Kazuma Yamagata, Risa Adachi, Koki Ishiyama, Kazuyoshi Kozawa, Kanna Nagaishi, Masaki Katayose, Keigo Taniguchi

**Affiliations:** ^1^ Department of Physical Therapy, School of Health Sciences, Sapporo Medical University, Sapporo, Japan, sapmed.ac.jp; ^2^ Graduate School of Health Sciences, Sapporo Medical University, Sapporo, Japan, sapmed.ac.jp; ^3^ Department of Anatomy, Division of Functional Morphology, School of Medicine, Sapporo Medical University, Sapporo, Japan, sapmed.ac.jp

**Keywords:** mechanical properties, muscle strain, stress–strain curve

## Abstract

Hamstring strain injuries frequently occur during high‐speed running, when muscle tension is markedly increased. Given the viscoelastic properties of muscle, stress during elongation is expected to be velocity‐dependent. Therefore, this study investigated the effect of elongation velocity on the stress in individual hamstring muscles. Seven Thiel‐embalmed cadaveric lower limbs were used to isolate the biceps femoris long head, semimembranosus, and semitendinosus muscles. Specimens were fixed to a testing apparatus and passively elongated to 10% strain at five elongation velocities (20–300 mm/min), while tensile load and displacement were recorded. Muscle cross‐sectional area was measured using ultrasound to calculate stress. A two‐way repeated‐measures analysis of variance examined the effects of muscle and velocity on stress at 10% strain. The results revealed no significant interaction between muscle and velocity (*p* > 0.05); however, both velocity and muscle had significant main effects on stress (*p* < 0.001). Post hoc analysis indicated that stress increased significantly with velocity (20 mm/min: 44.6 kPa, 50 mm/min: 47.4 kPa, 100 mm/min: 50.8 kPa, 200 mm/min: 56.4 kPa, 300 mm/min: 61.1 kPa). These findings suggest that stress in the hamstring muscles increases with elongation velocity, which may contribute to the risk of strain injuries during high‐speed running. However, the results should be interpreted in the context of cadaveric testing and elongation velocities that are substantially lower than those observed in vivo.

## 1. Introduction

Hamstring strain injuries are among the most common muscle injuries in sports, particularly during sprinting [1–5]. A systematic review reported that of 2761 documented hamstring strain injuries, 55.3% occurred during the late swing phase or immediately after foot contact [6]. These injuries primarily affect the biceps femoris long head muscle (BFlh) [2, 6]. The high incidence of injuries during sprinting and the frequent involvement of the BFlh suggest that biomechanical factors specific to this phase and muscle contribute to the injury mechanism.

During sprinting, hamstring muscle force increases, especially in the late swing phase [7–10]. Muscle force comprises both active and passive components [11] and serves as an important index in modeling and assessing muscle strength during sprinting [10, 12]. Studies using animal tissue have shown that muscles are most vulnerable to damage when actively contracting and lengthening, rather than passively lengthening [13, 14]. Accordingly, previous research has emphasized the active force generation of individual hamstring muscles [7–10]. However, interpreting findings across these studies requires caution. Fiorentino et al. demonstrated that faster running speeds increase muscle–tendon complex length, thereby elevating tensile forces [12]. In contrast, Thelen et al. found that increased running speed raises muscle tension without corresponding changes in muscle–tendon length [10]. These conflicting results suggest that factors beyond length changes—such as active force and muscle viscosity—may contribute to force increases at higher speeds. This underscores the need for further investigation into how these mechanisms differ among the hamstring muscles.

Whereas active force arises from muscle fiber contraction, passive force is generated by connective tissue resistance and plays a key role in muscle stiffness and elasticity. Because passive force affects muscle extensibility, load transmission, and resistance to stretch, understanding the passive properties of individual hamstring muscles is essential for evaluating muscle function and injury risk. Passive force is influenced by elongation‐dependent and velocity‐dependent factors [15], and previous studies have shown that responses to elongation vary among the hamstring muscles [16]. However, whether velocity‐dependent changes in passive tension differ among the hamstrings remains unclear. Although several studies have demonstrated that strain rate increases stress [15, 17–20], strain rate responses depend on specimen type, scale, and muscle properties [19]. The hamstrings include the semimembranosus (SM) and semitendinosus (ST) muscles in addition to the BFlh, and morphological characteristics such as cross‐sectional area, muscle mass, and fascicle length differ among them [16, 21–23]. These differences may influence how stress responds to velocity, yet no study has examined this in human hamstring muscle–tendon complexes.

Taken together, previous studies indicate that hamstring strain injuries are closely associated with high tensile loading during sprinting, particularly in the late swing phase, involving both active and passive mechanical factors. While active force generation during eccentric contraction has been extensively studied, the velocity dependence of passive mechanical behavior remains poorly understood. Furthermore, it is unclear whether velocity‐dependent passive stress responses differ among individual hamstring muscles, despite their known morphological and architectural differences. Clarifying these issues is essential for improving biomechanical understanding of hamstring strain mechanisms and for refining translational interpretations related to injury risk. Therefore, this study aimed to standardize the degree of elongation across the hamstring muscles and investigate the effect of elongation velocity on stress in individual hamstring muscles.

## 2. Materials and Methods

### 2.1. Preparation of Human Cadavers

Informed consent was obtained from the individuals and their families for participation in the body donation program (Shiragiku‐kai) managed by Sapporo Medical University, which supports medical and scientific research. This study utilized seven Thiel‐embalmed cadaveric specimens (five females and two males). At the time of death, the donors had a mean age of 88.8 ± 3.0 years, reported an antemortem height of 153.0 ± 3.3 cm, and reported an antemortem weight of 51.1 ± 5.4 kg. From each specimen, the BFlh, ST, and SM muscles were carefully dissected.

Thiel embalming uses a solution containing propylene glycol and a low concentration of formalin, which preserves tissue flexibility by preventing excessive hardening [24, 25]. Based on these findings, the mechanical behavior of the hamstring muscles observed in this study was considered to approximate key aspects of in vivo passive mechanical behavior under controlled conditions. This preservation technique is widely used in anatomical and biomechanical research because it allows repeated mechanical testing under controlled laboratory conditions. To minimize environmental effects on tissue properties, specimens were maintained in Thiel solution under controlled conditions (temperature: 22°C; relative humidity: ∼40%) throughout all procedures. This study was approved by the institutional ethics committee (approval number: 4‐1‐70) and conducted in accordance with ethical guidelines.

### 2.2. Experimental Protocol

**FIGURE 1:**
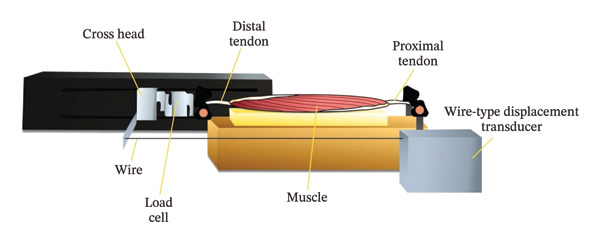
Experimental setup for velocity‐controlled passive elongation testing. Schematic illustration of the experimental setup used to assess velocity‐dependent passive stress in isolated hamstring muscle specimens.

An overview of the experimental setup and testing protocol is shown in Figure 1. The experiment followed a previously established protocol [16, 26], with the key modification that elongation velocity was systematically varied while strain was controlled. The proximal and distal tendons of each muscle were fixed using a parallel tightening jaw (width: 19 mm, height: 19 mm, opening width: 10 mm; JM‐JFM‐500N; A&D Co., Ltd., Tokyo, Japan). A tensile load was then applied to the distal tendon using a tensile tester (MCT‐1150; A&D Co., Ltd., Tokyo, Japan). Slack length (ML) was defined as the muscle length at the initial point of measurable loading. Prior to testing, the ML and anatomical cross‐sectional area (ACSA) at slack length were recorded. ACSA was measured in the muscle’s central region using B‐mode ultrasonography (Aixplorer version 12, MSK mode; Hologic, Marlborough, MA, USA).

**FIGURE 2:**
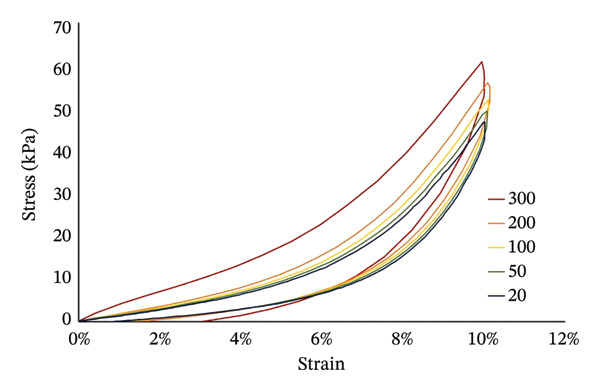
Representative examples of hysteresis loops at different elongation velocities: representative stress–strain hysteresis loops obtained during cyclic passive elongation at five testing velocities (20, 50, 100, 200, and 300 mm/min). The loops illustrate velocity‐dependent changes in the passive mechanical behavior of the hamstring muscle–tendon complex, with higher velocities resulting in increased stress and altered hysteresis characteristics. Axes are shown with representative quantitative scaling to facilitate interpretation of viscoelastic responses.

Following established tensile testing procedures for isolated muscle–tendon tissues, a preconditioning phase consisting of six loading cycles to 5% strain (half of the target strain) was conducted to stabilize the viscoelastic response prior to data collection [16, 27, 28]. A 60 s rest interval was incorporated between cycles to permit stress relaxation and reduce time‐dependent bias. After preconditioning, a single loading cycle to 10% strain (ΔL/L) was performed at each velocity condition, and stress (P/A) values at 10% strain were used for statistical analysis. Elongation was performed at five velocities: 20, 50, 100, 200, and 300 mm/min. For each velocity condition, slack length was identified as the onset of measurable load and used as the reference length (0% strain) for that condition. This specimen‐ and condition‐specific referencing was adopted to reduce the influence of slack variability on strain calculation when comparing stress at a constant target strain. The order of velocity application was randomized for each specimen to minimize potential cumulative viscoelastic effects. Representative hysteresis loops at different speeds are shown in Figure 2. The elongation velocities applied in this study ranged from 20 to 300 mm/min (approximately 0.33–5.0 mm/s), corresponding to nominal strain rates of approximately 0.001–0.015 s^−1^ based on the initial muscle lengths. This velocity range was selected to enable controlled and stable passive mechanical testing of the muscle–tendon complex. Although these velocities are substantially lower than the muscle lengthening rates reported during sprinting in vivo (e.g., ∼7900–9300 mm/s; [10]), they fall within the quasi‐static to intermediate strain‐rate range commonly used in soft tissue mechanical testing. Accordingly, the present protocol was designed to characterize velocity‐dependent passive stress behavior under controlled conditions rather than to directly replicate sprinting mechanics.

Throughout testing, specimens were kept moist with Thiel solution to minimize tissue dehydration. The total testing duration per specimen was approximately 40–60 min, including preconditioning cycles and all randomized velocity conditions.

### 2.3. Measurement of Displacement and Tensile Force

Displacement and tensile force were measured using a tensile tester (MCT‐1150; A&D Co., Ltd., Tokyo, Japan), a wire‐type displacement transducer (Special Order, 4Assist Inc., Tokyo, Japan), a load cell (LC1205‐K050; A&D Co., Ltd., Tokyo, Japan), and a digital indicator (TD‐700T; TEAC Co., Tokyo, Japan). The load cell was mounted to the crosshead and connected to the digital indicator to capture tensile load. The wire‐type transducer, fixed to the load cell, enabled concurrent measurement of displacement and force. Displacement was calculated from changes in wire position, with original ML measured using a tape measure. Electrical signals representing tensile load and displacement were recorded via an analog‐to‐digital converter (PowerLab ML880, AD Instruments Co., Ltd., Bella Vista, NSW, Australia) and stored using Chart software (LabChart 8, version 8.1.17, AD Instruments Co., Ltd., Bella Vista, NSW, Australia).

### 2.4. Data Analysis

Cross‐sectional images were analyzed using ImageJ v1.53k (National Institutes of Health, USA). The pixel count corresponding to a 1 cm scale on B‐mode ultrasound images was determined and used as a reference for ImageJ analysis. High‐echo outlines of the epimysium were selected using the polygon selection tool, and the enclosed region was defined as the ACSA. The mean ACSA from two independent measurements was used in stress calculations. Strain and stress values were extracted from displacement and tensile load data captured at 1 s intervals using Lab Chart 8. Strain was calculated as the relative change in muscle length from the original ML, and stress was defined as tensile load divided by the ACSA.

Statistical analyses were performed using SPSS Statistics software (version 28.0, IBM Corp., Armonk, NY, USA). Normality was evaluated with the Shapiro–Wilk test. A two‐way repeated‐measures analysis of variance (ANOVA) examined the effects of muscle and velocity on stress at 10% strain. When significant main effects were identified, Bonferroni‐corrected post hoc tests were applied. Statistical significance was set at 5%, and partial eta squared (ηp^2^) values were computed to assess effect sizes. ^2^. According to Cohen’s guidelines, ηp^2^ values of 0.01, 0.06, and 0.14 were interpreted as small, medium, and large effects, respectively [29].

## 3. Results

**FIGURE 3:**
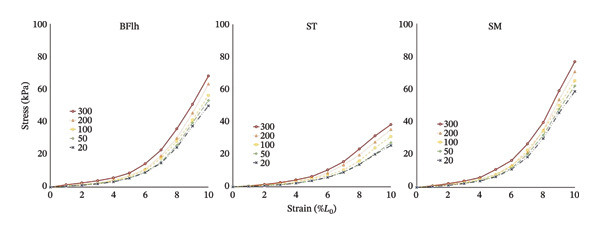
Velocity‐dependent changes in mean passive stress across hamstring muscles. Mean passive stress responses of the biceps femoris long head (BFlh), semimembranosus (SM), and semitendinosus (ST) across five elongation velocities. Color‐coded markers denote elongation speed: Red circles (300 mm/min), orange triangles (200 mm/min), yellow squares (100 mm/min), green diamonds (50 mm/min), and blue crosses (20 mm/min).

**FIGURE 4:**
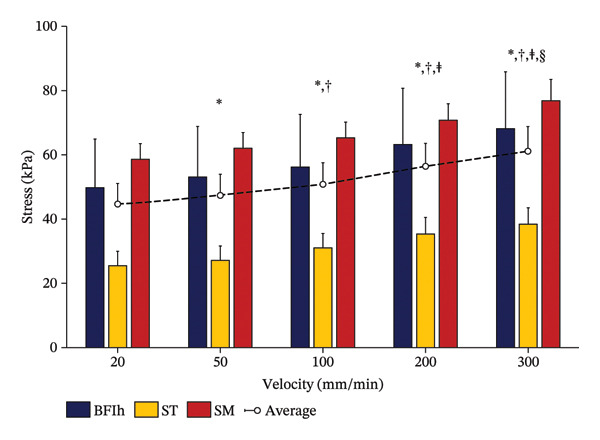
Comparison of passive stress among hamstring muscles at 10% strain passive stress values calculated at 10% strain for each hamstring muscle across all elongation velocities. Statistical comparisons indicate significant velocity‐dependent increases in stress, while differences among muscles reflect variations in absolute stress magnitude rather than velocity sensitivity. ^∗^ *p* < 0.05 vs. 20 mm/min; ^†^ *p* < 0.05 vs. 50 mm/min; ^‡^ *p* < 0.05 vs. 100 mm/min; ^§^ *p* < 0.05 vs. 200 mm/min.

Figure 3 illustrates the average change in stress for each hamstring muscle across the five velocity conditions (see Supporting Table 1 for complete data). At 10% strain, ANOVA showed no significant interaction between muscle and velocity (F_8,48_ = 1.370, *p* = 0.234, ηp^2^ = 0.186). However, significant main effects were observed for velocity (F_4,24_ = 227.644, *p* < 0.01, ηp^2^ = 0.974) and muscle (F_2,12_ = 7.119, *p* = 0.01, ηp^2^ = 0.543). Bonferroni‐corrected post hoc comparisons for velocity are presented in Supporting Table 2 and demonstrating a significant increase in stress with increasing elongation velocity. Mean stress values were 44.6 ± 14.0 kPa at 20 mm/min, 47.4 ± 14.8 kPa at 50 mm/min, 50.8 ± 14.5 kPa at 100 mm/min, 56.4 ± 15.2 kPa at 200 mm/min, and 61.1 ± 16.5 kPa at 300 mm/min (Figure 4). Additionally, stress in the SM was significantly greater than in the ST (BFlh: 58.1 ± 6.7 kPa; ST: 31.4 ± 4.8 kPa; SM: 66.6 ± 6.4 kPa).

## 4. Discussion

### 4.1. Velocity‐Dependent Passive Stress Behavior of the Hamstring Muscle–Tendon Complex

This study was designed to provide an initial characterization of velocity‐dependent passive stress behavior in the human hamstring muscle–tendon complex under controlled experimental conditions. By systematically varying elongation velocity while controlling strain, this study extends prior strain‐based investigations by isolating velocity as an independent mechanical variable. Under these conditions, passive stress increased significantly with increasing velocity across all muscles, with a total mean increase of approximately 37%. Individually, mean stress increased by 37% in the BFlh, 50% in the ST, and 31% in the SM between the lowest and highest velocity conditions.

However, no significant interaction between muscle and velocity was observed, indicating that the velocity‐dependent increase in stress was similar among the three hamstring muscles. To our knowledge, this is the first study to investigate the effects of varying velocities on passive stress responses in the human hamstring muscle‐tendon complex.

### 4.2. Viscoelastic Mechanisms and Implications for High Strain‐Rate Loading

The observed velocity‐dependent increase in passive stress is consistent with the nonlinear strain‐rate sensitivity of viscoelastic tissues, which has been attributed to viscous components within skeletal muscle and its extracellular matrix [15, 17, 20]. Classical viscoelastic modeling frameworks, including quasi‐linear viscoelastic theory, describe tissue stress as the combined contribution of an elastic response and a time‐ or rate‐dependent viscous component, with the viscous contribution becoming increasingly influential as strain rate increases [15, 17]. Within this framework, even modest velocity‐dependent stress increases observed under quasi‐static conditions may scale disproportionately at the substantially higher strain rates encountered during sprinting [15, 17, 19]. Accordingly, the present findings should be interpreted as a foundational step toward understanding how passive stress responses scale with velocity, rather than as a direct simulation of sprinting mechanics.

It is important to clarify that the higher stress observed at greater elongation velocities primarily reflects strain‐rate–dependent stiffening of viscoelastic muscle–tendon tissue. In practical terms, greater load is required to reach the same strain level when elongation occurs more rapidly. This does not necessarily imply increased intrinsic tissue vulnerability at a given strain magnitude. Muscle strain injury is more commonly associated with excessive strain, often in combination with high active force production and neuromuscular fatigue [7, 8]. However, even when strain magnitude is held constant, increased passive stress contributes to the overall mechanical load experienced by the muscle–tendon unit. Therefore, velocity‐dependent stress amplification may represent an additional loading factor during high‐speed movements rather than an isolated indicator of tissue susceptibility.

### 4.3. Muscle‐Specific Differences in Absolute Stress and Potential Injury Mechanisms

Although relative velocity sensitivity was similar across muscles, absolute stress magnitudes differed, with the SM exhibiting significantly higher stress than the ST, whereas no significant differences were observed between the BFlh and the other muscles. This pattern suggests that, under conditions of passive over‐elongation without active contraction, the SM may be subjected to a greater baseline mechanical load than the ST [16, 28]. This observation is consistent with experimental and clinical reports indicating that SM injury can occur under excessive stretch, even in the absence of pronounced muscle activation [30]. However, in vivo studies have also reported substantial SM activation during the late swing phase of running, indicating that this muscle is not purely passively loaded under physiological conditions [31, 32]. Therefore, the relative contributions of passive stretch‐related stress and active force generation to SM injury mechanisms warrant further investigation.

### 4.4. Passive Versus Active Contributions to BFlh Injury Risk

In contrast, although the BFlh is the most frequently injured hamstring muscle during sprinting, its passive stress did not differ significantly from that of the ST under the present experimental conditions [6–10]. This finding suggests that the high injury incidence of the BFlh cannot be fully explained by passive mechanical properties alone. Rather, injury susceptibility in this muscle likely emerges under dynamic conditions in which high strain rates coincide with substantial active eccentric force production [31]. Previous in vivo and modeling studies have demonstrated marked increases in BFlh force during high‐speed running, particularly during the late swing phase, when active lengthening predominates [6–10]. Taken together, these findings indicate that although velocity‐dependent passive stress responses are similar across hamstring muscles, muscle‐specific injury mechanisms likely depend on the interaction between baseline passive stress, active force generation, and task‐specific loading conditions.

### 4.5. Clinical Implications and Limitations

The present findings provide a biomechanical rationale for considering elongation velocity as a relevant factor influencing hamstring loading during athletic tasks. During high‐speed running, the hamstring muscles undergo substantial lengthening in the late swing phase, when both muscle–tendon lengthening velocity and mechanical demand are elevated [8, 10]. In this context, velocity‐dependent passive stress may act in parallel with active eccentric force to increase the overall mechanical load experienced by the hamstring muscle–tendon complex. From a clinical and practical perspective, these results suggest that rehabilitation and injury prevention programs should not rely solely on strength gains achieved under slow or quasi‐static conditions. Because all three hamstring muscles exhibited comparable velocity‐dependent increases in passive stress, interventions aimed at modifying passive mechanical behavior should consider the hamstring muscle group as a functional unit rather than focusing on individual muscles in isolation. Eccentric strengthening exercises, such as the Nordic hamstring exercise, are widely supported as effective strategies for reducing hamstring strain injury incidence [33–35]. However, emerging evidence indicates that isolated eccentric training and sprint‐based loading induce partly distinct neuromuscular and architectural adaptations [36, 37]. Therefore, combining eccentric strengthening with graded exposure to high‐speed running may be necessary to enhance tolerance to velocity‐ and strain‐rate–dependent mechanical loading encountered during sport‐specific activities.

Collectively, these findings suggest that hamstring injury risk may be influenced not only by muscle‐specific structural properties but also by velocity‐dependent passive stress acting in combination with active force production and neuromuscular factors [38, 39]. Accordingly, rehabilitation and injury prevention strategies should emphasize eccentric strengthening approaches that progressively restore tolerance to high elongation velocities encountered during sprinting and similar athletic tasks [40, 41].

Some limitations should be considered when interpreting these findings. First, the maximum velocity used in this study (300 mm/min = 5 mm/s) was substantially lower than the muscle lengthening velocities reported during sprinting in vivo. Prior studies have reported hamstring lengthening velocities of approximately 9300 mm/s during sprinting at 9.3 m/s [10], making our test velocity roughly 1860 times slower. Given the strain‐rate dependence of viscoelastic behavior, muscle‐specific differences in stress responses may emerge at higher velocities. Second, although Thiel‐embalmed specimens preserve tissue flexibility and are widely used in anatomical and biomechanical research, their mechanical properties may not fully replicate those of living or fresh‐frozen tissue. Previous investigations have reported mixed findings regarding the preservation of absolute biomechanical properties following Thiel fixation. While some studies have demonstrated comparable elastic modulus and tensile strength between Thiel‐preserved and fresh or fresh‐frozen specimens [42, 43], others have reported altered stiffness and elastic modulus in Thiel‐embalmed soft tissues [44]. These differences have been attributed to potential changes in tissue hydration, ionic composition, and protein crosslinking associated with the embalming process. Accordingly, although Thiel preservation allows consistent and reproducible passive mechanical testing under controlled laboratory conditions, the absolute stress magnitudes reported in the present study should not be interpreted as direct representations of in vivo material properties. Rather, the findings should be understood as reflecting velocity‐dependent trends in passive viscoelastic behavior under standardized cadaveric conditions. Third, our analysis focused on isolated hamstring muscles, while in vivo function involves interactions with adjacent muscle groups [45]. The absence of surrounding musculature and joint constraints in our experimental setup may limit the direct applicability of these findings to dynamic human movement. Fourth, the advanced age of the donor specimens (mean 88.8 years) should be considered when interpreting the findings. Aging has been associated with structural remodeling of muscle–tendon tissues, including alterations in extracellular matrix composition and increased collagen cross‐linking, which may influence passive mechanical behavior [46–48]. Such age‐related changes may affect absolute stress magnitudes and potentially strain‐rate sensitivity. Consequently, the passive mechanical responses observed in this elderly cohort may differ quantitatively from those of younger athletic populations in whom hamstring strain injuries most commonly occur. Finally, although the sample size (*n* = 7) is consistent with prior cadaveric biomechanical studies, specimen availability limits generalizability [16, 28, 49]. Nevertheless, the repeated‐measures design reduced interspecimen variability and enhanced statistical sensitivity. Future studies combining higher strain rates, in vivo measurements, and computational modeling are needed to further elucidate the role of velocity‐dependent passive stress in hamstring strain injury mechanisms.

## 5. Conclusion

This study demonstrated that stress in the hamstring muscles increases significantly with elongation velocity, indicating that passive muscle resistance is influenced by strain rate. However, no significant differences in velocity‐dependent stress responses were found among BFlh, SM, and ST, suggesting similar viscoelastic properties across the muscles under passive conditions.

**Nomenclature**
BFlh: Biceps femoris long head
SM: Semimembranosus
ST: Semitendinosus

## Author Contributions

Gakuto Nakao designed the study, contributed to the main conceptual ideas, and provided an outline of the proof. Kazuma Yamagata, Risa Adachi, Koki Ishiyama, and Kazuyoshi Kozawa collected the data. Kanna Nagaishi, Masaki Katayose helped in interpreting the results and worked on the manuscript. Keigo Taniguchi supervised the project. Gakuto Nakao wrote the manuscript with the support of Keigo Taniguchi.

## Funding

This study was supported in part by a grant‐in‐aid from the Japan Society for the Promotion of Science (grant number 23K10404 and 25K20965).

## Disclosure

All authors have approved the submitted version of the manuscript and agreed to be accountable for any part of this work, ensuring that questions related to the accuracy or integrity of any part of the study were appropriately investigated and resolved.

## Ethics Statement

All experimental protocols were approved by the Medical Research Ethics Committee of Sapporo Medical University (approval number: 4‐1‐70).

## Consent

The individuals and their families consented to participate in the body donation program (Shiragiku‐kai) run by Sapporo Medical University for medical and scientific research.

## Conflicts of Interest

The authors declare no conflicts of interest.

## Supporting Information

Additional supporting information can be found online in the Supporting Information section.

Supporting Table 2. Bonferroni‐corrected pairwise comparisons among elongation velocity conditions.

Pairwise comparisons of elongation velocity for passive stress at 10% strain (collapsed across muscles) derived from repeated‐measures ANOVA. Values represent estimated marginal mean differences (kPa), standard errors (SE), Bonferroni‐adjusted *p* values, and 95% confidence intervals.

## Supporting information


**Supporting Information 1** Supporting 1. Supporting Table 1. Descriptive statistics of passive stress at 10% strain across muscles and elongation velocities.

**Supporting Information 2** Supporting 2. Values are presented as mean ± standard deviation (kPa) for each muscle at each elongation velocity (20, 50, 100, 200, and 300 mm/min). These values correspond to the data used in the repeated‐measures ANOVA.

## Data Availability

The data that support the findings of this study are available from the corresponding author upon reasonable request.
